# *Leishmania braziliensis* isolated from disseminated leishmaniasis patients downmodulate neutrophil function

**DOI:** 10.1111/pim.12620

**Published:** 2019-04-14

**Authors:** Thiago Cardoso, Caroline Bezerra, Lilian Silva Medina, Rajendranath Ramasawmy, Albert Scheriefer, Olívia Bacellar, Edgar M. de Carvalho

**Affiliations:** ^1^ Laboratório de Pesquisas Clínicas (LAPEC) do Instituto Gonçalo Moniz – Fiocruz Bahia Brazil; ^2^ Serviço de Imunologia Hospital Universitário Prof. Edgard Santos Universidade Federal da Bahia Salvador Brazil; ^3^ The National Institute of Science and Technology in Tropical Diseases (INCT‐CNPq) Salvador Brazil

**Keywords:** cutaneous leishmaniasis, disseminated leishmaniasis, *Leishmania braziliensis*, neutrophils

## Abstract

**Aims:**

The polymorphism observed in *Leishmania braziliensis* is associated with different clinical forms of leishmaniasis. Neutrophils (PMNs) participate in the pathogenesis of leishmania infection, and here, we evaluate neutrophil function after infection with isolates of *L. braziliensis* from cutaneous leishmaniasis (CL) or disseminated leishmaniasis (DL) patients.

**Methods and results:**

Neutrophils from 30 healthy subjects (HS) were infected with isolates of *L*. (*V*.) *braziliensis* obtained from three CL and three DL patients. They were infected at the ratio of 3:1 parasites per neutrophil, and leishmania uptake was evaluated by microscopy. The neutrophil activation markers and oxidative burst by expression of dihidrorhodamine (DHR) were evaluated by flow cytometry and cytokine production by ELISA. The frequency of infected cells and the number of amastigotes were higher in neutrophils infected with CL isolates compared to DL isolates (*P* < 0.05). The DHR and CD66b expression after infection with DL isolate was lower than with CL isolates. There was no difference regarding chemokine production.

**Conclusion:**

The *L. (V.) braziliensis* isolates of DL induced lower respiratory burst and neutrophils activation markers compared with CL isolates which may contribute to parasite survival and dissemination in DL patients.

## 1 INTRODUCTION

The American tegumentary leishmaniasis (ATL) is caused predominantly by *L. (V.) braziliensis*, a parasite that is associated with different clinical forms of the disease as cutaneous, mucosal and disseminated cutaneous leishmaniasis (DL).^1^, ^2^ The cutaneous leishmaniasis (CL) is the most common presentation of the disease occurring in over 90% of the cases and is characterized by a well‐limited skin ulcer with raised borders. Approximately 3% of CL patients develop concomitantly or months and sometimes years after the cutaneous disease, mucosal leishmaniasis (ML) that affect primarily the nasal mucosa.^3^ DL is an emerging clinical form of ATL defined by the presence of ten up to more than 1000 acneiform, papular and ulcerated lesions in at least two parts of the body.^4^ In the majority of the cases, DL patients present initially as a typical CL ulcer, and after 1 or 2 weeks or sometimes even during antimony therapy, patients suddenly develop multiple lesions over the body. There are several indicators of the relevance to study this atypical presentation of ATL. DL is an emergent form of leishmaniasis as its prevalence increased 20‐fold from 1986 to 2012,^5^ up to 40% of DL patients have ML 4, 6 and the disease is associated with a high hate of failure to antimony therapy.^7^ The pathogenesis of DL is not fully understood. Initial studies showed that DL patients have more negative leishmania skin delayed type hypersensitivity test (LST) and an impairment in lymphocyte proliferation and in the production of interferon‐γ and TNF in supernatants of mononuclear cells stimulated with soluble leishmania antigen (SLA) as compared to CL patients.^8^ However, more recently, we document that cell‐mediated immune response at the lesion site of DL patients is similar to what is observed in CL ulcers.^6^, ^9^ We believe that there is no impairment in T‐cell response in DL patients and raised the hypothesis that the poor T‐cell response observed in cells from peripheral blood was due to the migration of the majority of to the antigen reactive cells to the great number of lesions observed in DL patients.^6^ Moreover, these data suggest that parasite, more than host factors, may be involved in the pathogenesis of DL.

It is known that *L. (V.) braziliensis* is polymorphic and genotypic differences intraspecies of *L. (V.) braziliensis* are associated with different clinical forms of ATL.^10^, ^11^ Specifically, it has been shown that there are six haplotypes presented in four loci of the chromosome 28 of *L. (V.) braziliensis* that are associated with DL.^10^ It has been also documented that some haplotypes in the chromosome 28 are also associated with failure to antimony therapy, indicating the importance of differences intraspecies not only in the presentation of the disease, but also in failure to therapy.^12^, ^13^ However, there is a lack of data about the biological behaviour of isolates from DL on phagocytic cells. Neutrophils migrate quickly to the sites of infection and are the main phagocytic cells in the initial phase of *L. (V.) braziliensis* infection.^14^ Studies in experimental models of leishmaniasis have shown that interaction of neutrophils with macrophage may determine the control or progression of leishmania infection.^15^ In humans, we have previously shown that neutrophils from CL produce higher levels of reactive oxygen species and pro‐inflammatory cytokines than healthy subjects neutrophils upon *L. (V.) braziliensis* infection.^16^

To determine whether the behaviour of *L. (V.) braziliensis* isolated from patients with DL differs from that observed with parasites from CL patients, we evaluate in the present study the ability of *L. (V.) braziliensis* isolates from DL and CL patients to penetrate or be up‐taken by neutrophils from healthy subjects, as well as whether the genotypic differences among these isolates would influence the neutrophil activation and leishmania killing.

## 2 MATERIALS AND METHODS

### 2.1 Subjects

Venous blood was obtained from 30 healthy volunteers from a nonendemic area of leishmaniasis. They denied previous history of leishmaniasis, and none of them have a scar of CL. The group was composed of 17 males and 13 females, and the mean age was 27 ± 12 years. The participants did not have previous contact with *Leishmania*, and they did not present other infectious diseases and denied symptoms of viral infections and fever at the time of peripheral blood sample collection. The study was approved by Institutional Review Boards (IRBs) of the Federal University of Bahia (Ethical Committee), and informed consent was obtained from all participants.

### 2.2 Isolation of human peripheral blood neutrophils

Peripheral blood was collected in EDTA, and cells were isolated from the blood using PMN™ Isolation Medium (Axis‐Shield PoC AS, Oslo, Norway). The purity of granulocytes was always >97% as determined by microscopic evaluation after May‐Grunwald‐Giemsa staining from cytocentrifuge preparations. The viability of cells was >98% as assessed by tripan blue dye exclusion. The time between bleeding and infection was about 50 minutes (30 minutes of separation and 10 minutes for wash the cells twice with 0.9% NaCl).

### 2.3 Parasites

*L*. (*V*.) *braziliensis* genetically distinct^10^, ^17^ isolated from CL and DL patients were used for neutrophil infection. The isolates were obtained from three CL patients, and three DL patients diagnosed in Corte de Pedra, Bahia, Brazil, an area of *L. (V.) braziliensis* transmission. The CL was defined by an ulcerated skin well‐limited lesion with raised borders in the absence of mucosal involvement.^9^ DL was defined as the presence of 10 or more mixed acneiform, papular and ulcerated lesions located in two or more different parts of the body.^4^ The diagnosis of leishmaniasis was confirmed by documentation of DNA of *L. (V.) braziliensis* in tissue biopsy specimens.^15^ Further DNA from *L. (V.) braziliensis* was genotyped according to the nucleotide polymorphic alleles detected at the chromosome locus CHR28 at initial position 425451, previously shown to distinguish strains of *L. (V.) braziliensis* in Corte de Pedra‐BA.^10^ A pair of 5′‐primers, TAAGGTGAACAAGAAGAATC and 5′‐CTGCTCGCTTGCTTTC, was used to amplify a 621 nucleotide segment in CHR28 / 425451 from *L. (V.) braziliensis* DNA.^13^

### 2.4 Neutrophils infection

The isolates of 03 CL and 03 DL patients were used to infect PMNs of 30 healthy subjects participants of the study. For each experiment, neutrophils from one healthy subject were infected, separately, with each one of the 3 isolates of CL and DL. Briefly, promastigotes forms of *L. (V.) braziliensis* from CL and from DL patients were grown in Schneider axenic medium and were used in stationary growth phase. Suspensions from LIT/NNN were transferred to Schneider's medium with 10% heat‐inactivated foetal calf serum and 2 mM L‐glutamine, and incubated at 26°C until they reached a density of 10^7^ cells/mL. Neutrophils, 3 × 10^6^ in 1 mL of RPMI 1640 medium (Gibco BRL, Grand Island, NY, USA), supplemented with 50 μM 2‐mercaptoethanol, 2 mM L‐glutamine, 10 mM HEPES, antibiotic and 10% of foetal bovine serum (Gibco BRL) and were incubated, at 37°C, with *L. (V.) braziliensis* (promastigotes on stationary phase) at ratio of 3:1 neutrophils for 30, 90 and 180 minutes. After these periods, the number of infected cells and the number of intracellular parasites were determined by microscopic in 200 cells stained with Grunwald‐Giemsa on cytocentrifuge microscopy slides preparations.

### 2.5 Assessment of respiratory burst of neutrophils

The production of reactive oxygen species (ROS) was evaluated by flow cytometry using the fluorogenic substrate dihydrorhodamine 123 (DHR 123, Cayman Chemical Company, Ann Arbor, MI, USA) as an indicator of production of ROS. Briefly, 5 × 10^5^ neutrophils were incubated with 1 μM of DHR123 for 15 minutes and after infected with *L. (V.) braziliensis* promastigotes or PMA (10 ng/mL) plus ionomycin (500 ng/mL) or buffer was added. Control samples containing no stimulus were runs in parallel. After 15 minutes at 37°C, 5% CO_2_, cells were washed in PBS and analysed by flow cytometry. The neutrophil population was selected based on forward and side scatter followed by DHR123 fluorescence to determine the frequency and mean of fluorescence intensity (MFI) of double positive cells CD15^+^DHR^+^. Separate controls verified of this population, corresponded to neutrophils according to CD15^+^ surface stain. The FACS strategy to assessment of respiratory burst is shown in Figure 2A.

### 2.6 Assessment of neutrophils activation markers by flow cytometry analysis

CD62L and CD66b are known to be, respectively, downregulated and upregulated on PMNs upon stimulation/activation.^18^ PMNs uninfected and infected with *L*. (*V*.) *braziliensis*, or stimulated with ionomycin (500 ng/mL) *plus* 10 ng/mL of phorbol‐myristate‐acetate (PMA), were cultured for 90 minutes at 37°C and 5% CO_2_. Cells were stained with PE‐conjugated MAb α‐CD62L, APC‐conjugated MAb α‐CD66b and PE‐Cy7‐conjugated MAb α‐CD15 (BD Pharmigen). PMNs were stained with MAbs in diluent buffer (20 minutes at 4°C) washed two times with PBS 1X and then fixed with 2% formaldehyde. About 20 000 events were gated, acquired by FACS Calibur (Becton Dickinson and Company, Franklin Lakes, NJ, USA) and analysed by Flow Jo Software (Becton Dickinson). The frequency of double positive cells CD62L^+^/CD15^+^ and CD66b^+^/CD15^+^, and the mean of fluorescence intensity (MFI) were determined followed FACS strategy shown in Figure 3A.

### 2.7 Evaluation of CXCL8 and CXCL9 production

The supernatants of uninfected neutrophils, cells infected with different *L. (V.) braziliensis* isolates or stimulated with ionomycin/PMA were obtained after 90 minutes and frozen at −20°C. The production of CXCL8 and CXCL9 was measured by enzymatic immune assay (ELISA) (R&D Systems, Minneapolis, MN, USA) according to the manufacturer's instructions.

### 2.8 Statistical analysis

The Friedman's test was used to analyse the frequency of infected neutrophils as well as the parasite burden, obtained by count of two independent blind analysers. The frequency of cells expressing activation molecules (CD62L and CD66b) and the mean fluorescence intensity (MFI) were analysed by Kruskal‐Wallis followed by Dunn's post‐test. These tests were also used to evaluate the expression of DHR on CD15^+^ cells and IL‐8 production. Analyses were performed using Prism GraphPad software. The *P*‐value of <0.05 was considered significant.

## 3 RESULTS

### 3.1 Frequency of infected Neutrophils and parasite load after infection with different isolates of *L. (V.) braziliensis*

**Figure 1:**
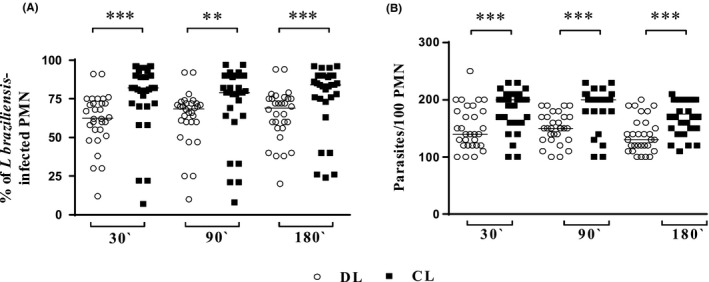
Uptake of DL and CL *L. (V.) braziliensis* isolates by healthy subjects PMN's. Neutrophils from healthy subjects (n = 30) were incubated with stationary phase *L. (V.) braziliensis* isolates (three isolates from DL and three isolates from CL) at a 3:1 parasite/neutrophil ratio, under conditions that allow phagocytosis. After 30, 90 or 180 min of incubation at 37°C, 5% CO_2_, cytocentrifuge slides were prepared and stained with Giemsa. The frequency of infected neutrophils (A) and the number of intracellular parasites per 100 neutrophils (B) were determined microscopically, analysed by two double‐blind surveys. Each symbol represents the mean value of the infection observed with three isolates of DL (○) or CL (■) in neutrophils from different subjects. Lines represent the median of the data. Statistical analyses were performed using the Friedman's test (**P < 0.01,***P < 0.001)

The Figure 1A,B shows the frequency of infected neutrophils and the number of intracellular amastigotes per 100/cells and its point represents the mean of the data obtained with 3 isolates of CL and DL in cells of each participant of the study. The frequency of infected neutrophils at 30 minutes by DL isolates was lower (62.5 ± 17.4%) compared to CL isolates (82.0 ± 23.0%), at 90 minutes (68.5 ± 18.0% *vs* 79.0 ± 24.3%) and at 180 minutes (69.0 ± 16.8% *vs* 84.0 ± 21.8%) *P* ≤ 0.01 (Figure 1A). The parasite load of infected neutrophils was expressed as number of internalized parasites per 100/cells. The number of amastigotes per 100 cells after 30 minutes of infection was lower with DL isolates (140 ± 38) than with CL isolates (200 ± 35) *P* ≤ 0.01. After 90 minutes of infection, the number of amastigotes in cells infected with isolates from DL was 150 ± 26 vs 202 ± 35 in cells infected with the CL isolates, *P* ≤ 0.001, and after 180 minutes, the parasite burden was 130 ± 30 vs 175 ± 30 respectively, *P* ≤ 0.001 (Figure 1B).

### 3.2 Reactive oxidants production by infected neutrophils with different *L. (V.) braziliensis* isolates

**Figure 2:**
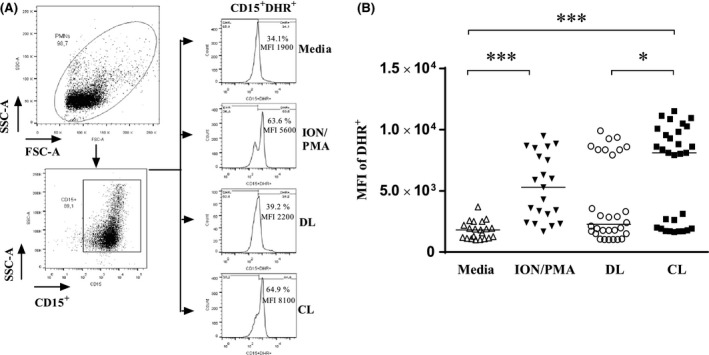
Release of reactive oxidants by neutrophils from healthy subjects induced by phagocytosis of *L. (V.) braziliensis*. A, Representative FACS strategy dot‐plots indicating the SSC‐A vs FSC‐A gated population and purity of PMN cells based CD15^+^ expression. Representative histograms showing DHR‐123 fluorescence due to reactive oxidants in unexposed neutrophils from a healthy subject, or the same neutrophils exposed to Ion/PMA and *L. (V.) braziliensis* isolates. B, Graphical presentation of the MFI of DHR‐123 staining in neutrophils under the same conditions. Statistical analyses were performed using the Kruskal‐Wallis test followed by Dunn's post‐test (*P < 0.05, ***P < 0.001). The values shown are representative of five independent experiments with 30 healthy donors and were express in median

We also evaluated the capacity of different isolates of *L. (V.) braziliensis* to trigger oxidant production in neutrophils. Fluorescence of DHR‐123, an indicator of the abundance of cellular reactive oxidants, was measured by flow cytometry. The Figure 2 presents the MIF of CD15^+^DHR^+^ uninfected cells (medium) and infected neutrophils. Ionomycin *plus* PMA was used as positive control. Uninfected cells expressed a little of ROS, and the median of MIF of DHR expression was similar in uninfected cells and in neutrophils infected with isolates of DL (2274). In contrast to neutrophils infected with CL isolate, the median of DHR expression was 8110 (*P* ≤ 0.05). The reactive oxidants production measured by MIF of the median of DHR expression in cells infected with *L. (V.) braziliensis* from DL (2274) was similar to that detected in cells cultured with media and lower than that observed with isolates from CL (8110), *P* ≤ 0.05 (Figure 2B).

### 3.3 Neutrophils activation induced by *L. (V.) braziliensis* infection

The CD62L and CD66b expressions are widely used to detect neutrophil activation. CD62L is an integrin shed from neutrophil surfaces upon activation,^18^, ^19^, ^20^, ^21^ and decreased expression of this molecule indicates neutrophil activation. We evaluate whether different isolates of *L. (V.) braziliensis* activate the neutrophils and the Figure 2A shows the MIF of CD62L on uninfected and infected neutrophils. Ionomycin *plus* PMA was used as positive control. There was no difference in the intensity of fluorescence expression of CD62L between neutrophils infected with DL isolate or CL isolate (Figure 2B). However, the frequency of CD15^+^CD62L^+^ cells was higher on neutrophils infected with DL isolate (38 ± 16%) than that observed with CL isolates (19 ± 14%), *P* ≤ 0.05.

**Figure 3:**
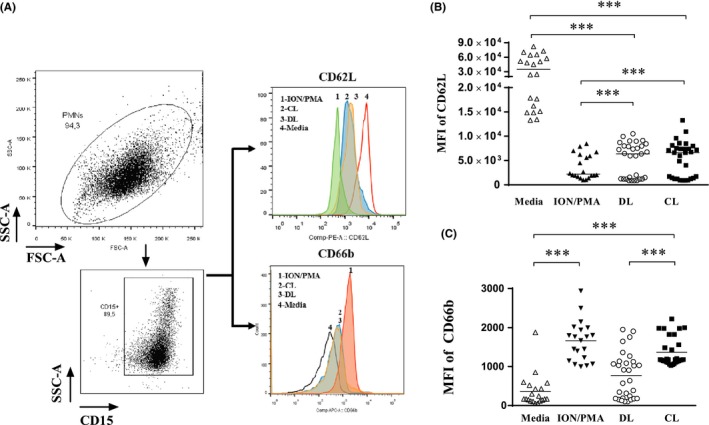
Effect of *L. (V.) braziliensis* infection on expression of neutrophil's activation markers. (A) Representative FACS strategy dot‐plots indicating the SSC‐A vs FSC‐A and purity of PMN cells. Histograms demonstrating neutrophils CD15^+^ CD62L^+^ and CD15^+^ CD66b^+^ population under unexposed, stimulated with Ion/PMA and infected with *L. (V.) braziliensis* isolates. Collated results of surface staining for activation markers by flow cytometry expressed in MFI of CD62L (B) and CD66b expression (C) over neutrophil's surface. Each value represents the MFI of activation markers of one healthy subjects’ neutrophils. Statistical analysis was performed using the Kruskal‐Wallis test followed by Dunn's post‐test, comparing stimulated to infected and unstimulated cells (***P < 0.001). The values shown are representative of five independent experiments with 30 healthy donors and were express in median

CD66b is neutrophil granule membrane proteins that migrate in the surface membrane upon granule exocytosis.^22^ Neutrophils infected with DL isolates expressed less CD66b on cells surface than neutrophils infected with CL isolate, *P* ≤ 0.001 (Figure 3C).

### 3.4 IL‐8 and CXCL9 production by PMNs after infection with different *L. (V.) braziliensis* isolates

**Figure 4:**
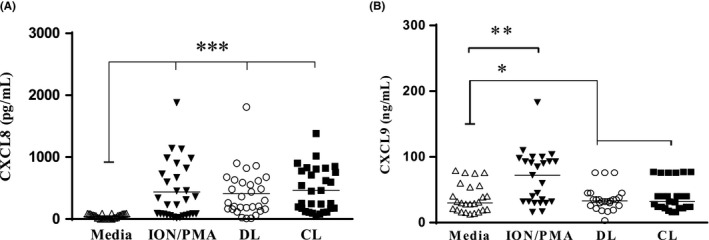
Chemokines produced by healthy subjects PMNs after infection with DL and CL *L. (V.) braziliensis* isolates: PMNs culture supernatants (n = 30) were submitted to ELISA for CXCL8 (A) and CXCL9 (B) measurement. The concentrations of chemokines were evaluated and analysed using the Kruskal‐Wallis test followed by Dunnn's post‐test (*P < 0.001). Each symbol represents mean values from different subject neutrophils, and lines represent the median of each group

The cytokines IL‐8 and CXCL‐9 were measured in supernatants of neutrophil 90 minutes after infection with *L. (V.) braziliensis* obtained from DL or CL patients. The production of IL‐8 did not differ in supernatants of PMNs infected with isolates of *L. (V.) braziliensis* from DL or CL patients (*P* > 0.05). Production of CXCL‐9 was lower than IL‐8 but follows the same pattern with no difference in supernatants of cells infected with DL or CL isolates (data not shown). There was no difference between IL‐8 (Figure 4A) and CXCL‐9 (Figure 4B) production in cultures infected with CL or DL isolates production of these cytokines were high and similar was observed in cells stimulated with Ion/PMA.

## 4 DISCUSSION

DL is an emerging and severe form of *L. (V.) braziliensis* infection associated with a high rate of ML and failure to antimony therapy.^4^, ^7^ Substantial variability among the leishmania at the subgenus level has been described, and there are evidences that genotypic differences intraspecies may influence the presentation of the disease^10^ and response to therapy.^13^ However, the biological behaviour of *L. (V.) braziliensis* isolates with distinct genetic characteristics in immune cells of the host is necessary to better understand how the parasites influence disease outcome. *L. (V.) braziliensis* from DL patients are less up‐taked and or penetrate less in neutrophils, induce lower respiratory burst as well cellular activation markers than isolates from CL.

The role played by neutrophils in the pathogenesis of leishmania infection has been only recently emphasized. We knew previously that PMNs may internalize and kill leishmania; however, as macrophages are the host cells for this parasite, most of the studies have evaluated the consequences of the interaction of macrophages with leishmania.^23^, ^24^ Neutrophils are the first cell type to migrate to the site of leishmania infection^25^ and the interaction of infected neutrophils with macrophages may attenuate or worse the pathology in mice with different genetic background.^26^ Comparing the internalization of *L. (V.) braziliensis* in neutrophils from CL patients with cells from HS, we found that *L. (V.) braziliensis* is taken up by both CL and HS neutrophils at similar rates.^16^ Here using HS cells, we compared the internalization of *L. (V.) braziliensis* obtained from a DL patients with isolates from CL patients and found at all times points that the frequency of infected neutrophils was higher in neutrophils infected with parasites obtained from CL than parasite from a DL patients. The number of intracellular amastigotes of *L. (V.) braziliensis* from isolates of DL was also lower than that observed with isolates from CL patients. However, as the number of amastigotes was similar at all time points, apparently, there was no difference in parasite replication between CL and DL isolates. This indicates that isolates from DL penetrate less efficiently in neutrophils than *L. (V.) braziliensis* from CL patients.

The phagocyted microbes are destroyed by oxidative and nonoxidative mechanisms inside the neutrophils cytoplasm. The uptake of microbes induces the oxidative burst, increases reactive oxygen species (ROS) production and this leads to parasites clearance.^27^, ^28^ Monocytes produce ROS after exposure to *L. (V.) braziliensis,* and in this cell, ROS contribute to control parasite multiplication.^29^, ^30^ In contrast to monocytes, the ROS generated by infected neutrophils prevents parasite multiplication but did not decrease the number of intracellular amastigotes.^16^ This may explain why despite the lower oxidative burst induced by isolates of DL in comparison with CL isolates, the number of intracellular parasites was similar at all time points with different *L. (V.) braziliensis* isolates. One limitation of the present study was the use of EDTA as anticoagulant. One previous study showed that the use of EDTA rather than heparin or citrate decreases neutrophil activation after stimulation with PMA.^31^ However, as in all experiments of our study, EDTA was used the differences observed were related in the source of *L. braziliensi*s isolates rather than methodological aspects.

Following infection or exposure to PMA neutrophils increases the CD66b expression and decreases CD62L expression, surface markers indicative of an activated phenotype.^19^, ^20^ The CD66b expression indicates exocytosis from specific granules, and the decreased expression of CD62L is indicative of an increased ability of the cell to migrate out of the circulation.^21^ We have previously shown that neutrophils from both CL and HS infected with an isolate of *L. (V.) braziliensis* obtained from a CL patient are similarly activated.^16^ Here, we showed that neutrophils infected with a DL isolate expressed less CD66b than cells infected with CL isolates. While we cannot ruled out that the lower number of intracellular parasites observed in neutrophils infected with DL isolates may have influenced a decreasing in the expression of neutrophils activation markers and in the oxidative burst, these data clearly indicate that genotypic differences among *L. (V.) braziliensis* isolates modify neutrophil function.

The CD66b is endogenous in specific granules, and its increased appearance on the neutrophils surface indicates exocytosis from specific granules.^19^ The release of proteolytic enzymes, defences and myeloperoxidase from intracellular granules into phagosomes independently cooperates with neutrophil function to enhance microbicidal activity.^14^, ^32^ Our observation that isolates from DL induced less CD66b expression on PMN than isolates from CL indicates that parasites from DL decrease the release of granules decreasing neutrophil function.

The CD62L is a homing receptor that is cleaved from neutrophils surface upon activation, and its loss facilitates cell migration out of the circulation.^21^ Neutrophils are the first cell to arrive in the leishmania penetration site. The MFI expression of CD62L was similar in neutrophils infected with a CL and DL isolates. However, as the frequency of cells infected with DL expressing CD62L was lower than the frequency of cells infected with a CL isolate, we cannot ruled out that *L. (V.) braziliensis* from DL patients by decreasing the number of cells expressing CD62L decrease neutrophil migration to the lesion site. If this is the case, it may facilitate the penetration of *L. (V.) braziliensis* in macrophages that is the most important host cell for leishmania.

Neutrophils produce a variety of cytokines and chemokines including IL‐12, CXCL‐8, CXCL‐9, CXCL‐10, CCL‐3, CCL‐4, IL‐23 and IFN‐γ.^33^ We have previously shown that neutrophils from CL patients upon *L. (V.) braziliensis* infection produce more CXCL‐8 and CXCL‐9 than neutrophils from HS.^16^ Here using HS neutrophil, we observed that IL‐8 and CXCL‐9 production did not differ in supernatants from cells infected with isolates of CL or isolates of DL patients. These data indicate that although *L. (V.) braziliensis* from a DL patient penetrate less in neutrophil and decrease neutrophils activation markers, these isolates did not impair chemokine production. This observation also argues against the possibility that parasite from DL induced lower respiratory burst and lower neutrophil activation than isolates from CL because they are less internalized by PMNs.

DL is a very severe form of *L. (V.) braziliensis,* and studies have already clinically defined the disease. However, little is known about how parasite may disseminate. We have previously found no impairment in the T‐cell response of DL patients. Here, we show that isolates from DL behaved differently than CL isolates in neutrophils. This find gives support to the hypothesis that genotypically different isolates of the same leishmania species induce different immune responses which may influence disease expression.

## DISCLOSURES

All authors of this manuscript deny any conflict of interest.

## Data Availability

The data that support the findings of this study are openly available in figshare at https://figshare.com/s/db4f2afd54d0c7e95a79]. Data citation: Thiago Cardoso; 2019; *Leishmania braziliensis* Isolated from Disseminated leishmaniasis Patients Down Modulate Neutrophil Function.
